# Integration of single-cell sequencing and bulk expression data reveals chemokine signaling pathway in proliferating cells is associated with the survival outcome of osteosarcoma

**DOI:** 10.1186/s12920-023-01617-5

**Published:** 2023-08-03

**Authors:** Lin Yu, Sun Hongyu, Chen Yuxi

**Affiliations:** 1 Department of Neurology, The First Affiliated Hospital of Harbin Medical University, Harbin, Heilongjiang China; 2 The Second Affiliated Hospital, Qiqihar Medical University, Qiqihar, Heilongjiang China; 3 Department of Orthopedic Surgery, The First Affiliated Hospital of Harbin Medical University, Harbin, Heilongjiang China

**Keywords:** Osteosarcoma, Chemokine signaling pathway, Proliferating cells, Biomarker

## Abstract

**Background:**

Osteosarcoma, as the most common primary bone malignancy, is urgent to be well-studied on the biomarkers and therapeutic targets to improve the five-year survival rate. Transcriptomic analysis using single-cell RNA or bulk RNA sequencing has been developed to detect biomarkers in various cancer types.

**Methods and results:**

We applied Scissor to combine single-cell RNA-seq data and bulk transcriptome data of osteosarcoma, providing cell-level information and sample phenotypes to identify the survival-associated cell subpopulations. By investigating the differences between the survival-associated cell subpopulations, we identified CCL21, CCL22, CCL24, CXCL11, CXCL12, CXCL13, GNAI2, and RAC2 in the proliferating cells that are significantly associated with osteosarcoma patient outcome. Then we assigned the risk score for each sample based on the cell proportion-normalized gene expression and validated it in the public dataset.

**Conclusions:**

This study provides the clinical insight that chemokine signaling pathway genes (CCL21, CCL22, CCL24, CXCL11, CXCL12, CXCL13, GNAI2, and RAC2) in proliferating cells might be the potential biomarkers for treatment of osteosarcoma.

**Supplementary Information:**

The online version contains supplementary material available at 10.1186/s12920-023-01617-5.

## Introduction

Osteosarcoma is the most common primary bone malignancy, deriving from primitive bone-forming mesenchymal cells [1]. The two main age groups of osteosarcoma patients are 10–14-year-old teenagers and adults older than 65 years old [1]. Although the therapeutic methods for various types of cancer have been significantly developing, the 5-year survival rate of 60% has not improved since the mid-1980s [2]. One main reason is that biomarkers and targets for osteosarcoma have yet to be well studied [3]. Therefore, it is critical to identify biomarkers with clinical insights to guide treatment and improve the survival rate of osteosarcoma patients.

Taking advantage of RNA sequencing (RNA-seq) for bulk tissues, some research groups have discovered some clues for biomarkers of osteosarcoma. For example, the overexpression of FGFR1 and downregulation of CHM may be the treatment target for osteosarcoma patients [4]. In addition to the protein-coding genes, some non-coding genes have been revealed as potential markers, such as microRNA miR-214-3p [5] and long non-coding RNA SNHG3 [6].

However, sequencing data of bulk tissues considers the whole tissue's averaged properties, disregarding the influence of the various cell types for the complex intro-tumoral heterogeneity and the tumor microenvironment in osteosarcoma. Recently, single-cell RNA-seq (scRNA-seq) was developed to allow zoom-in pictures of diverse cell types and cross-talk between cells in a heterogeneous tissue ecosystem [7]. Yet scRNA-seq is practical in small sample sizes, which brings difficulties in identifying the specific phenotype-associated cell subpopulations and biomarkers. Leveraging the phenotype information of the bulk RNA-seq study with large cohorts to guide the identification of the subpopulation and biomarkers showed an excellent insight into detecting the highly disease-relevant cell subsets [8].

In this study, we applied the integration of scRNA-seq data and bulk RNA expression data to explore the biomarkers associated with patient prognosis and play a vital role in the development of osteosarcoma. According to the different gene expression patterns of cell types, we identified the proliferating cells and the corresponding differentially expressed genes associated with patient survival outcomes. To deconvolute the bulk gene expression that measured the average level of the mixture of multiple cells, we used the differentially expressed genes in each cell type as markers to infer the cell proportions of each cell type of bulk samples. The cell proportion-normalized gene expression could be the potential biomarker to predict the patient survival outcome. With the cross-validation by the public datasets, our results detected a group of potential biomarkers in the chemokine signaling pathway of proliferating cells that are closely related to patient survival outcomes, which may improve the diagnostics and prognosis of osteosarcoma.

## Materials and methods

### Single-cell RNA-seq dataset collection and processing

The scRNA-seq dataset was downloaded from NCBI Gene Expression Omnibus (GEO) database under the accession number GSE152048 [7]. Cells that with lower than 200 or higher than 5,000 expressed genes were removed. We further discarded cells with mitochondria content higher than 10%. Finally, 105,740 cells were obtained for the downstream analysis. Batch effects within the samples were removed using the integration process of the Bioconductor/R package Seurat (v4.0.6) [9]. First, normalize the expression using the function NormalizeData with the default parameters (normalization.method = "LogNormalize", scale.factor = 10,000). Then, use the FindVariableFeatures with default parameters to identify the top 2,000 variable genes and perform principal component analysis. The first 30 principal components were used to determine the clusters with 0.5 resolution settings, obtaining 19 clusters—the first 30 principal components to were used to visualize the whole dataset.

Based on the canonical cell markers, we assigned 19 clusters into 17 different cell types, including fibroblasts (FBLN1), myeloid cells (CD74, CD14, FCGR3A), chondroblasts cells (SOX9, ACAN, PTH1R), osteogenic cell (IFITM5), proliferating cells (MKI67, TOP2A, PCNA), osteoclasts (ACP5, CTSK, MMP9), T cells (CD3, IL7R, CD8A, CD4, NKG7), peripheral monocyte cell (S100A8, FCN1), endothelial cells (PLVAP), osteocyte (DMP1), pericytes (RGS5, ACTA2), osteoblastic cells (IBSP), macrophage (SPI1), inflammation osteoblastic cells (IFT3, IFT1, IFT2, RSAD2), mesenchymal stem cells (MME, THY1, CXCL12, SFRP2), dendritic cells (IRF8, GZMB, JCHAIN), and mast cells (TPSAB1, CPA3, TPSB2, MS4A2).

### Bulk RNA expression datasets collection

One bulk RNA expression dataset was downloaded from the GEO database under the accession number GSE21257 [10]. The clinical information of 53 samples and probe ID conversion were retrieved from the matrix file using a self-code Python script. The sample clinical information included age, gender, histological subtype, tumor location, huvos grade, survival time, and survival status.

The other bulk RNA expression dataset was collected from the project Therapeutically Applicable Research To Generate Effective Treatments (TARGET) Osteosarcoma of The Cancer Genome Atlas Program (TCGA). The gene expression and the survival information of 88 samples were downloaded using the Bioconductor/R packages TCGAbiolinks (v2.20.1) [11].

### Identification of survival-associated cell subpopulations and the corresponding specific expressed genes

The Bioconductor/R package Scissor (v2.0.0) [8] was used to identify the survival-associated cell subpopulations. Following the instruction of Scissor, we prepared the expression matrix of the bulk RNA dataset (GSE21257), the corresponding single cell expression matrix (GSE152048), and the survival information of the samples in the bulk RNA-seq data with default parameters of Scissor to identify cells that were positive- and negative-associated with prognosis. To verify the reliability of positive- and negative- results, we also performed the “Reliability significance test”.

FindMarkers function of the Seurat package was used to identify the differentially expressed genes between the different cells within one cell type. The differentially expressed genes with adjusted *P*-value < 0.05 and |log2FoldChange|> 1 were assigned as significantly differentially expressed genes.

### Functional enrichment analysis

The enrichment analysis of the differentially expressed genes was performed using the Bioconductor/R package clusterProfiler (v4.3.1) [12], on GO [13], KEGG [14–16], and WikiPathway [17]. Functions with adjusted *P*-value < 0.05 were considered the significantly enriched functions.

### Cell proportion inferring in bulk expression datasets

The integrated expression of cell markers from different cell types and the top 20 differentially expressed genes identified by FindMarkers was defined as the signature of cell type expression. We used CIBERSORTx web-server [18] to deconvolute the bulk expression matrix into proportions of different cell types.

### Survival Analysis and risk score calculation

For each cell type, we extracted the expression of genes in each function pathway and normalized the expression by dividing the cell proportion. Then we used the Cox Proportional-Hazards Model of the Bioconductor/R package survminer (v0.4.9) to evaluate the beta values of each gene. The summed-up weighted gene expression in each function pathway was used to represent the risk score. The average of the risk scores was used as the threshold to distinguish the high- and low-risk samples.

### Visualization

All the visualization were performed using ggplot2 (v3.3.5) (https://github.com/tidyverse/ggplot2), clusterProfiler (v4.3.1), Seurat (v4.0.6), ggpubr (v0.4.0.999) (https://github.com/kassambara/ggpubr), survminer (v0.4.9) (https://github.com/kassambara/survminer), enrichplot (v1.12.3) (https://github.com/YuLab-SMU/enrichplot), and EnhancedVolcano (v.13.2) (https://github.com/kevinblighe/EnhancedVolcano) in the R environment (v4.0.0).

### Codes available

All the processing codes used in this paper were available on the zenodo.org (10.5281/zenodo.7982216).

## Results

### Cell atlas of human osteosarcoma

We performed scRNA-seq analysis on the public osteosarcoma dataset to obtain the osteosarcoma's cell type composition profile. After removing the low-quality cells and doublets, we finally obtained a single-cell transcriptome from 105,740 cells. We then removed the batch effects, as severe batch effects were observed in the dataset (Figure S1).

**Fig. 1:**
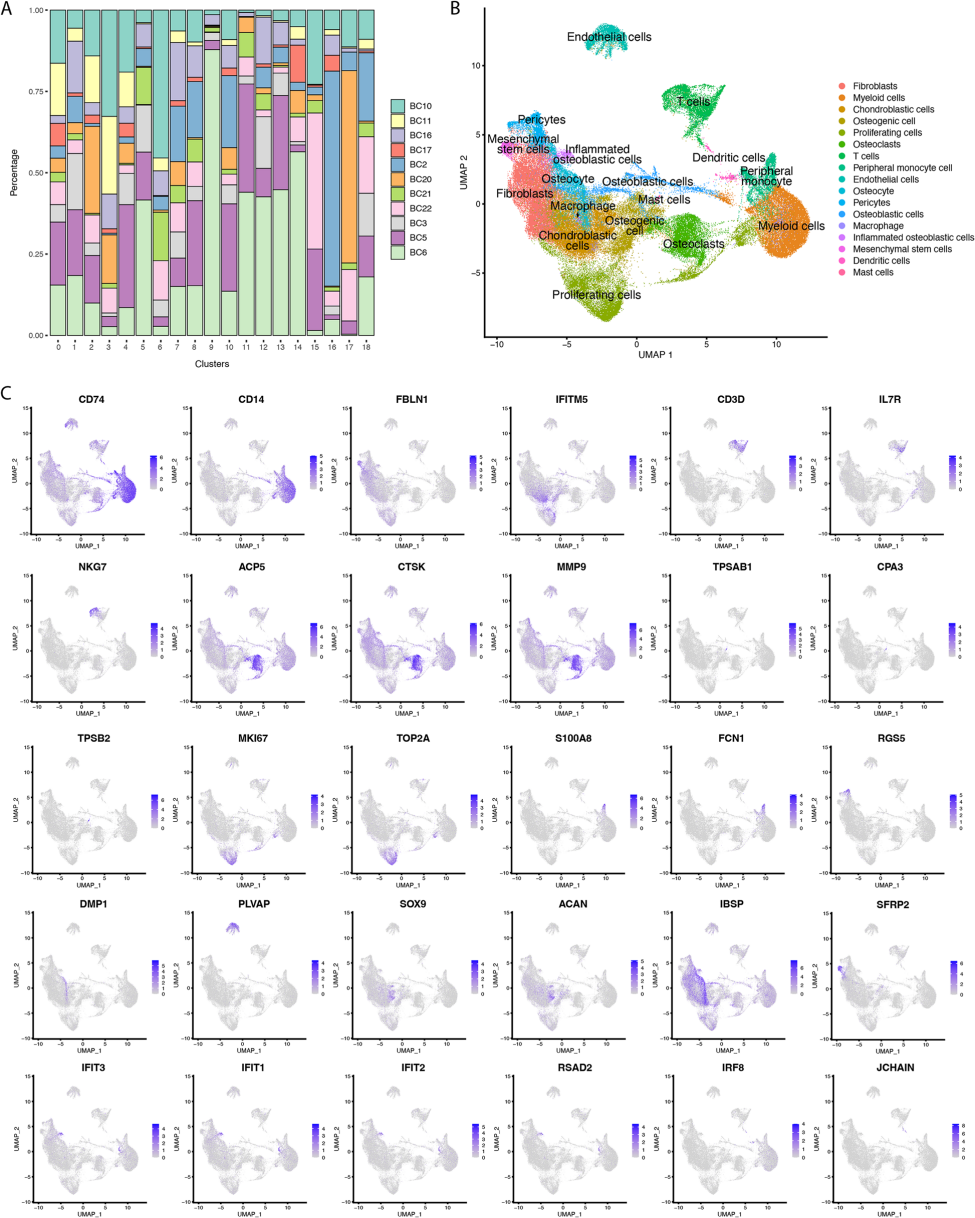
Distinct clusters of cells in osteosarcoma. **A** Stacked bar-plot showed the cell compositions of different samples in each cluster. **B** UMAP plot of the de-dimensional osteosarcoma scRNA-seq dataset. **C** Feature plots for marker genes of each cell cluster

After the batch effects canceling, we identified 19 clusters and assigned them to 17 cell types according to the canonical markers (Fig. 1A-B). Compared to the original study [7], six more cell types were identified. In our identification: (1) the fibroblasts highly expressed FBLN1; (2) the myeloid cells expressed CD74, CD14, and FCGR3A; (3) the chondroblastic cells highly expressed SOX9, ACAN, and PTH1RP; (4) the osteogenic cell expressed IFITM5; (5) the proliferating cells expressed MKI67, TOP2A, and PCNA; (6) the osteoclasts expressed ACP5, CTSK, and MMP9; (7) the T cells expressed CD3, IL7R, CD8A, CD4, and NKG7; (8) the peripheral monocyte cell expressed S100A8 and FCN1; (9) the endothelial cells expressed PLVAP; (10) the osteocyte expressed DMP1, (11) the pericytes expressed RGS5 and ACTA2; (12) the osteoblastic cells expressed IBSP; (13) macrophage expressed SPI1; (14) the inflammated osteoblastic cells expressed IFT3, IFT1, IFT2, and RSAD2; (15) the mesenchymal stem cells expressed MME, THY1, CXCL12, and SFRP2; (16) the dendritic cells expressed IRF8, GZMB, and JCHAIN; (17) the mast cells expressed TPSAB1, CPA3, TPSB2, and MS4A2. The expression profiles of these markers in the cell populations were demonstrated in Fig. 1C and Figure S2.

The endothelial cells were relatively isolated from other cell types in the Uniform Manifold Approximation and Projection (UMAP) de-dimension plot, which showed similar features to the previous study [7]. The cluster 11 and 17 both represented osteoblastic cells, but the composition of the samples was different in them. In cluster 11, the predominant samples were BC5 and BC6, which were conventional tumor samples. In contrast, in cluster 17, the dominant samples were BC20 and BC22, which are chondroblasts tumor samples, indicating the heterogeneity of osteoblastic cells.

### Survival-associated cell subpopulation profile in osteosarcoma

**Fig. 2:**
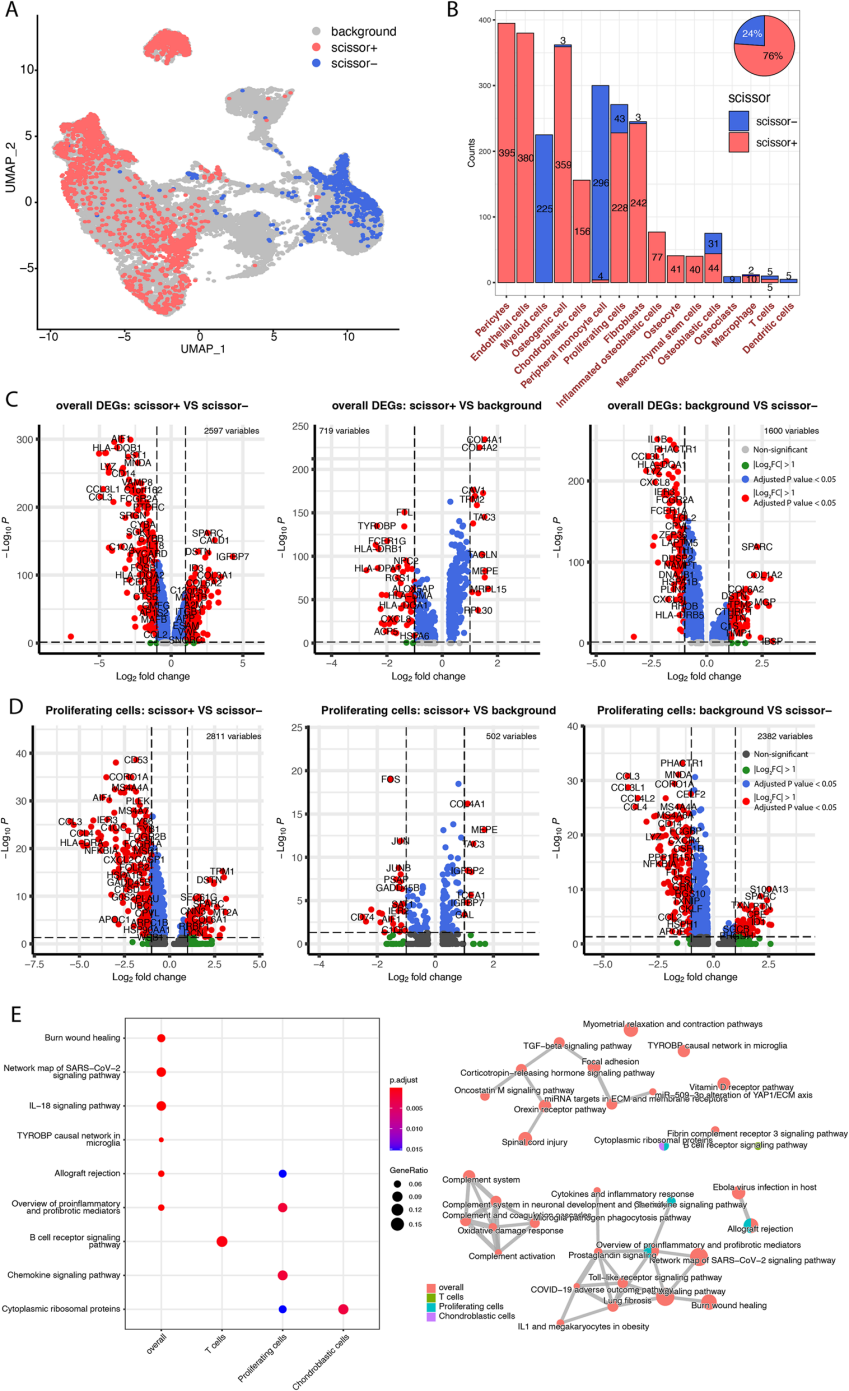
Survival-associated cell subpopulations, genes, and pathways. **A** UMAP plot of the identified survival-associated cell subpopulations. Scissor + (red) cells represent associating with worse survival; Scissor- (blue) cells represent associating with better survival. **B** Stacked bar-plot of the survival-associated cell distribution in each cell type. Y-axis represents the cell count number; X-axis represents cell types. The pie chart (top right) represents the global ratio of scissor + and scissor- cells. **C**-**D** The volcano plots of differentially expressed genes in overall cells (**C**), and proliferating cells (**D**). From left to right are considering scissor + vs. scissor-, scissor + vs. background, and background vs. scissor-. The red dot represents significantly differentially expressed genes with adjusted *P* value < 0.05 and |log2FoldChange|> 1. Dots in grey, green, and blue represent non-significantly differentially expressed genes. **E** Functional enrichment analysis of the differentially expressed genes. The dot-plot on the left panel describes the top five enriched items in WikiPathway. The network on the right panel represents the connection between the WikiPathways. The shared pathways were colored according to cell types. The connection represents the shared gene between the two pathways. In the left dot-plot, the enrichments gene ratio was proportional to the circle size, and the color represents different significance. In the right network plot, the circle size is proportional to the pathway size, and the color orange represents overall enrichment results; the color green represents T cell enrichments results; the color blue represents Proliferating cell enrichment results; the color purple represents chondroblatic cells enrichments results

Using the Scissor, we identified 1,981 scissor + cells associated with worse survivals and 622 scissor- cells associated with good survivals (Fig. 2A), which has been processed reliability test according to the Scissor instruction and proved reliable (*p* < 0.0001). The scissor + cells occupied a more significant proportion of the associated cells (Fig. 2B, scissor + vs. scissor-: 76% vs. 24%) and were observed as distributed in 13 different cell types, dominated most cancer cells, including pericytes, endothelial cells, and osteogenic cell (Fig. 2B). The scissor- cells were observed as distributed in 10 different cell types, dominated by myeloid cells and peripheral monocyte cells (Fig. 2B). However, few immune cells (T cells and macrophages) were identified as significantly associated with prognosis. There was a comparable number of scissor + cells and scissor- cells in osteoblastic cells, indicating different subtypes within the osteoblastic cells.

Considering the transcriptome differences between scissor + cells, background cells (non-survival-associated cells), and scissor- cells, we found the SPARC, which is highly expressed in highly metastatic tumors [19], significantly up-regulated in scissor + cells when compared to scissor- cells. The same up-regulated was also observed in comparing background cells and scissor- cells, i.e., the SPARC expression was lower in the scissor- cells (Fig. 2C). In addition to the overall comparison, we also compared the differences between scissor + and scissor- cells in each cell type. In the proliferating cells, the SPARC was identified as having the same tendency as in the overall comparison, indicating the differential expression in proliferating cells (Fig. 2D). In the scissor + dominated cell type, fibroblasts, we identified FOS, JUNB, IER3, and GADD45B were down-regulated while TAGLN, ACTA2, MYL9, PRSS23, IFI27, and ISG15 were up-regulated (Figure S3A). In another scissor + dominated cell type, osteogenic cells (Figure S3B), JUNB was also down-regulated. The deduction of JUNB was reported to cause increasing proliferation and tumorigenicity in wild-type murine fibroblasts [20], which was consistent with our findings.

Next, we investigated the functions of the differentially expressed genes. The differentially expressed genes were enriched in immune-related pathways, including the TGF-beta signaling pathway, cytokines and inflammatory response, and allograft rejection (Fig. 2C). The overview of proinflammatory and profibrotic mediators was shared by both overall and proliferating cells enriched pathways. The cytoplasmic ribosomal proteins were shared by the proliferating cells and chondroblasts cells (Fig. 2C). The survival-associated genes within the proliferating cells were highly related to chemokine activity, cytokine activity, and CCR chemokine receptor binding pathways (Fig. 2E, Figure S4A, B), indicating the chemokine-related function activities were highly enriched in proliferating cells which contributed to better survival outcome.

### Deconvolution of the cell composition in bulk RNA expression data

**Fig. 3:**
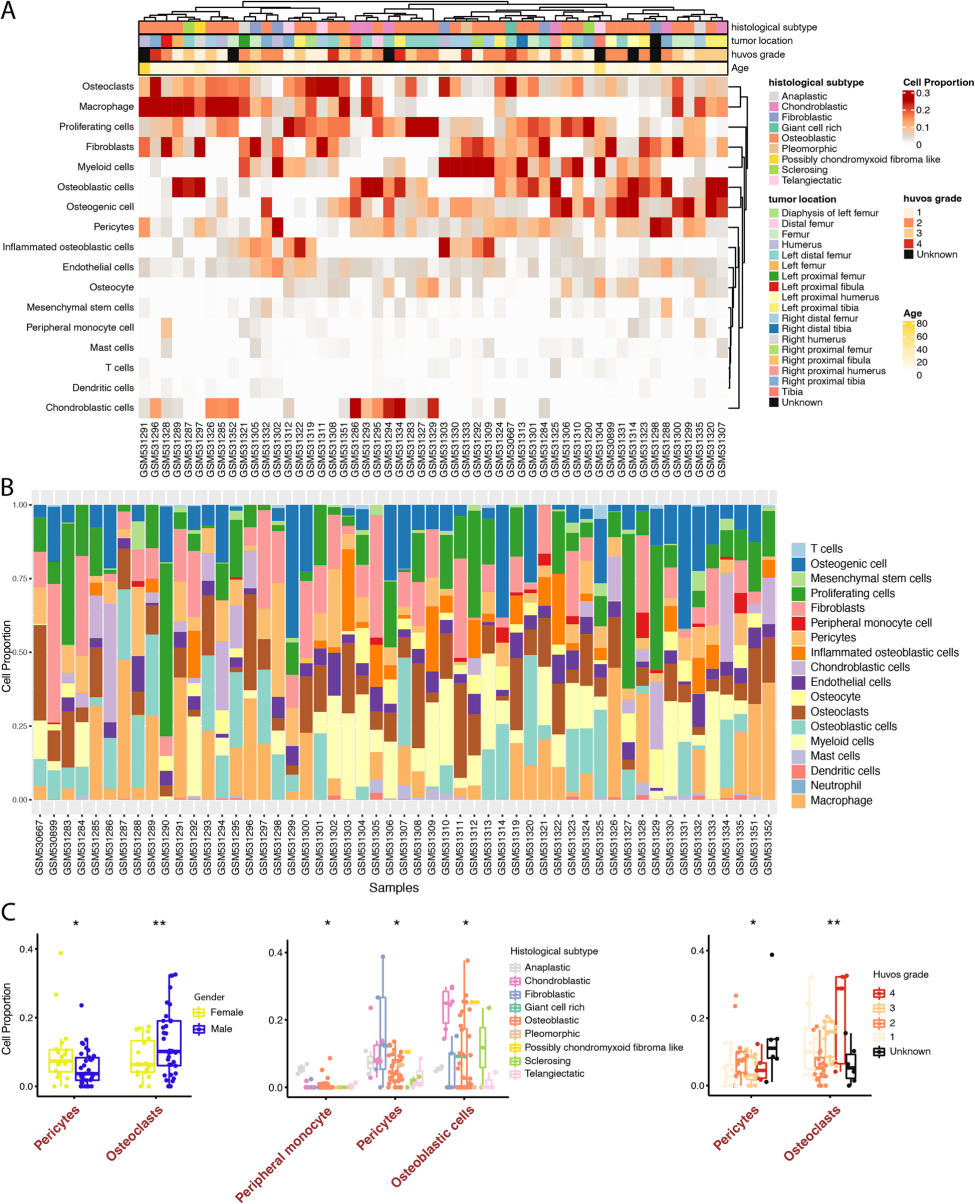
Diversity of cell proportions in osteosarcoma bulk transcriptome data. **A** Heatmap showed the cell proportion in the osteosarcoma bulk transcriptome data. The sample phenotype annotations, including histological subtype, tumor location, huvos grade, and age, were shown on the top of the heatmap. **B** Stacked bar-plot of the cell proportions in the osteosarcoma bulk transcriptome data. **C** Boxplots of cell proportions grouped by different sample phenotypes. From left to right are gender, histological subtype, and huvos grade. The x-axis represents cell types; the y-axis represents cell proportion. Significant symbol: *: *P* < 0.05, **: *P* < 0.01

To investigate the cell composition of the bulk RNA expression data, we referred to the CIBERSORTx algorithm to predict the proportion of different cell types in each sample based on RNA expression data. Macrophage, osteoclasts, proliferating cells, and fibroblasts occupied the most significant proportion of the samples, while osteocyte, T cells, mast cells, and peripheral monocyte cells existed less in the samples (Fig. 3A).

Cell proportions are distributed in different samples, indicating the differences in sample extraction and types (Fig. 3B). As the gene expression in bulk tissue represented the average expression of varying cell types weighted by cell proportions [18], determining the cell proportion should be an essential process in the transcriptome study.

Regarding the sample phenotypes, we also observed significant differences in cell proportion (Fig. 3C). The proportion of pericytes was the most fluctuated, showing the significant difference between genders (*P*-value = 4.7e-02), histological subtypes (*P*-value = 3.1e-02), and huvos grades (*P*-value = 2.9e-02).

### Cell proportion-normalized gene expression predicted the survival outcome of patients with osteosarcoma

**Fig. 4:**
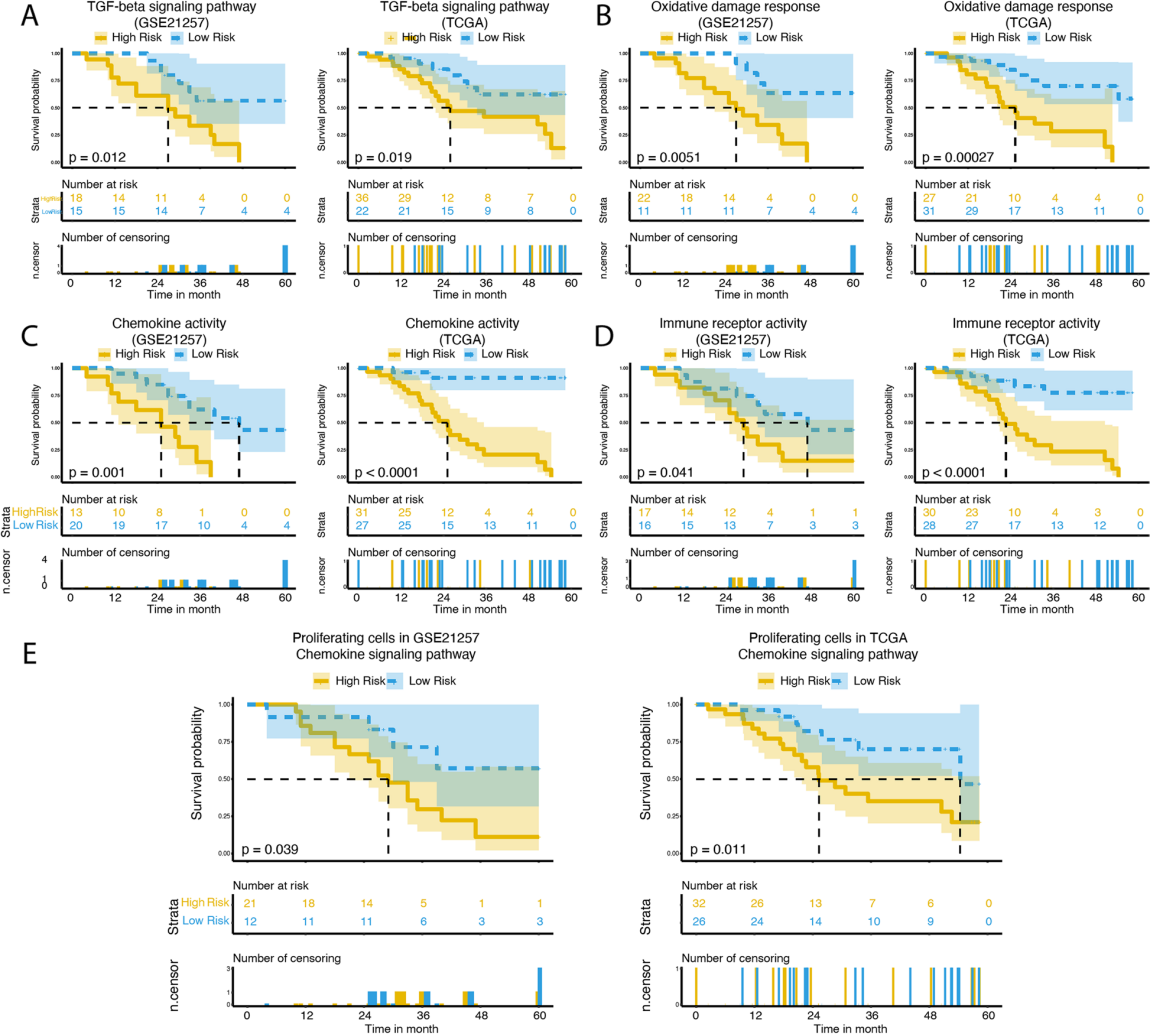
Kaplan–Meier curves for TGF-beta signaling pathway (**A**), oxidative damage response pathway (**B**), chemokine activity pathway (**C**), and immune receptor activity pathway (**D**) considering overall survival-associated genes. **E** Kaplan–Meier curves for chemokine signaling pathway considering proliferating cells survival-associated genes

We further characterized the prognostic potential of pathway genes. For the prognostic potential of the overall differentially expressed gene-enriched pathways, we determined the risk scores for each patient according to the gene expression and regression coefficients in the multivariate Cox model. The average value was used to classify the patients into low-risk and high-risk groups. Some pathways showed promising prognostic ability (Fig. 4A-D). The TGF-beta signaling pathway, which acted as tumor-suppressor functions including cell-cycle arrest and apoptosis [21], showed significant differences in the low-risk and high-risk groups. Besides the apoptosis-related pathway, oxidative damage response, chemokine activity pathway, and immune receptor activity pathway also showed as potential prognostic predictors.

In addition to the overall differentially expressed genes enriched pathways, we also investigated whether the cell-type specific enriched pathways could predict the prognostic outcome. We normalized the gene expression by the cell proportions. We found that the risk scores of the patients in the chemokine signaling pathway of proliferating cells were able to predict the patient outcome (Fig. 4E). The TCGA osteosarcoma dataset further confirmed the findings (Fig. 4A-E, Figure S5). These results suggested that the chemokine signaling pathway in proliferating cells could be used as a prognostic marker for osteosarcoma patients.

## Discussion

Osteosarcoma is a bone malignancy that attacks both teenagers and elders. The undeveloped five-year survival rate since the mid-1980s has promoted the exploration of diagnostic and therapeutic targets for osteosarcoma. In this study, with the leverage of clinical information from the bulk RNA project, we used the tool Scissor to guide the identification of the patient survival outcome-associated cell subpopulations and the differentially expressed genes in cells. To help reduce the cost of clinical testing in the future, we applied the biomarkers detected in the single-cell RNA dataset to the bulk RNA dataset, followed by normalizing the bulk gene expression using the corresponding cell proportions. With the validation in the public dataset, CCL21, CCL22, CCL24, CXCL11, CXCL12, CXCL13, GNAI2, and RAC2, enriched in the chemokine signaling pathway in proliferating cells, were determined as the potential biomarkers for treatment of osteosarcoma.

Chemokines are a group of small molecules that promote cell survival and proliferation, guiding the cell migration [22]. CCL21, CCL22, and CCL24 are genes encoded by C–C motif chemokine ligands, which are components of intercellular communication and essential in the functioning of the tumor microenvironment [23]. There have been promising findings showing that CC chemokines could be the target of cancer therapies, including colon carcinoma [24], lung cancer [25], ovarian cancer [26], and melanoma [27]. Similarly, CXCL11, CXCL12, and CXCL13 encoded the C-X-C motif chemokine ligands, critical regulators of tumor progression in many cancers [28–30]. Researchers have reported that CXCL12 is associated with the survival outcome in the osteosarcoma [31, 32]. Yet, only some studies focused on the potential clinical significance of other genes we identified in the chemokine signaling pathway, especially the predicting ability of the combination of these genes.

In conclusion, this study integrated the single-cell sequencing and bulk expression data and revealed that the chemokine signaling pathway in proliferating cells is associated with the survival outcome of osteosarcoma; however, it is essential to recognize the limitation of the study. Although we performed the validations using the TCGA dataset, no experimental validation was involved in this study. Further functional validations will be designed and implemented in future research. Nevertheless, compared to performing scRNA-seq or bulk RNA-seq to seek potential treatment targets for osteosarcoma, our study provided new insights into discovering survival outcome-associated biological pathways and biomarkers as therapeutic targets.

### Supplementary Information

**Additional file 1: Supplementary Figure S1.** Batch effect correction of the osteosarcoma scRNA-seq data. (A). UMAP plot and stacked bar-plot of the scRNA-seq data before removing the batch effects. (B). UMAP plot and stacked bar-plot of scRNA-seq data after removing the batch effects.

**Additional file 2: Supplementary Figure S2.** Violin plots showed cell marker expression in various cell types of the scRNA-seq data.

**Additional file 3: Supplementary Figure S3.** The volcano plots of the differentially expressed genes considering fibroblasts (A), osteogenic cells (B), and osteoblastic cells (C).

**Additional file 4: Supplementary Figure S4.** Functional enrichments analysis of Kyoto Encyclopedia of Genes and Genomes (KEGG) pathways (A) and Gene Ontology (GO) terms (B). The dot-plot on the left panel represents the top five enriched items in KEGG or GO. The network on the right panel represents the connection between the KEGG pathways or GO terms.

**Additional file 5: Supplementary Figure S5.** Stacked bar-plot of cell proportion in the osteosarcoma bulk transcriptome data from TCGA.

## Data Availability

Data are available in NCBI Gene Expression Omnibus (GEO) database under the accession number GSE152048 (https://www.ncbi.nlm.nih.gov/geo/query/acc.cgi?acc=GSE152048) and GSE21257 (https://www.ncbi.nlm.nih.gov/geo/query/acc.cgi?acc=GSE21257) without restrictions on the use or distribution of the data.
