# Supplementary figures and images for: Integration of single-cell sequencing and bulk expression data reveals chemokine signaling pathway in proliferating cells is associated with the survival outcome of osteosarcoma
Source: BMC Med Genomics. 2023 Aug 3;16:180. doi: 10.1186/s12920-023-01617-5 (PMC10399040; doi:10.1186/s12920-023-01617-5)

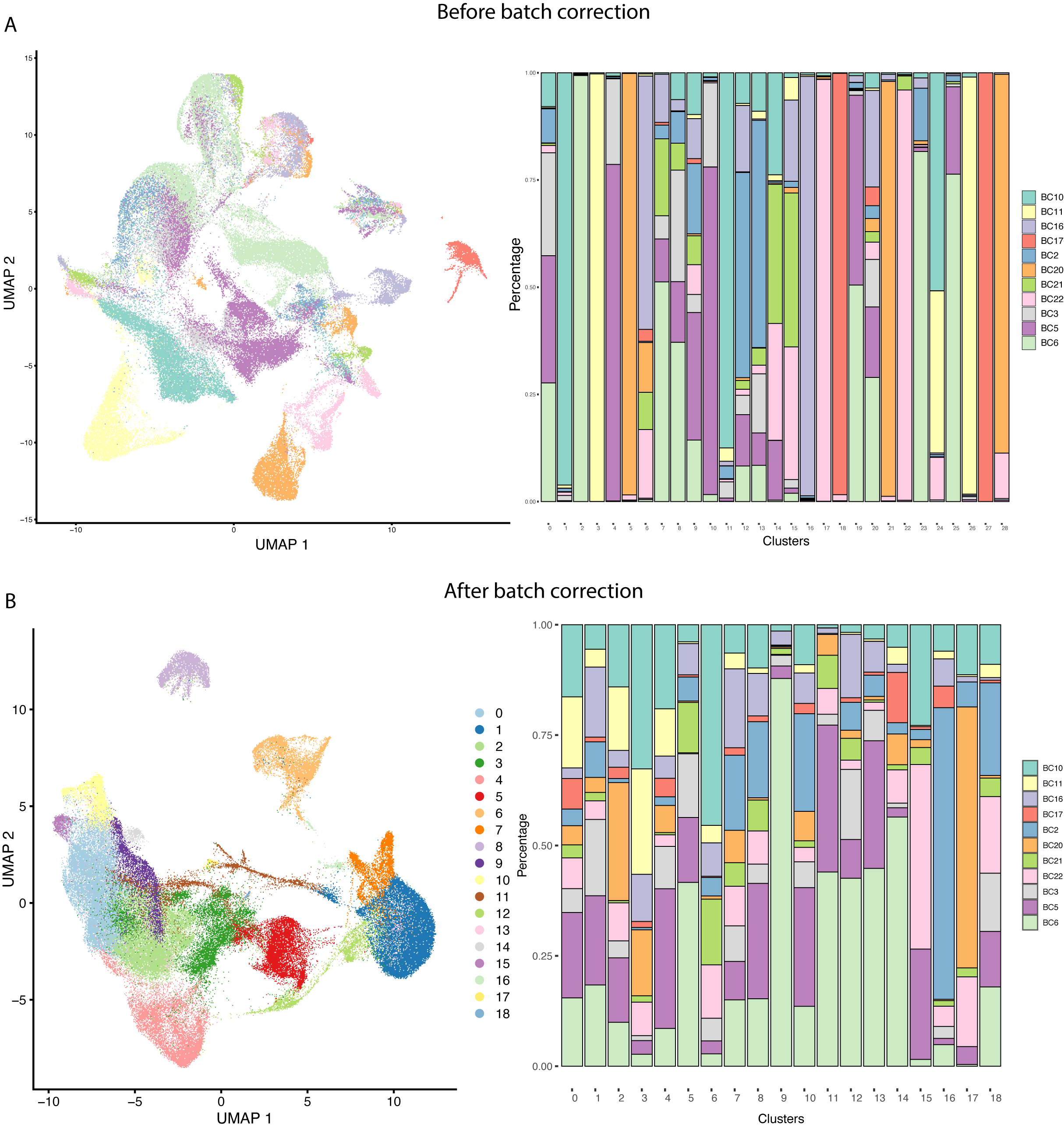

Supplement: Supplementary file 1 — Additional file 1: Supplementary Figure S1. Batch effect correction of the osteosarcoma scRNA-seq data. (A). UMAP plot and stacked bar-plot of the scRNA-seq data before removing the batch effects. (B). UMAP plot and stacked bar-plot of scRNA-seq data after removing the batch effects. [file 12920_2023_1617_MOESM1_ESM.tif]

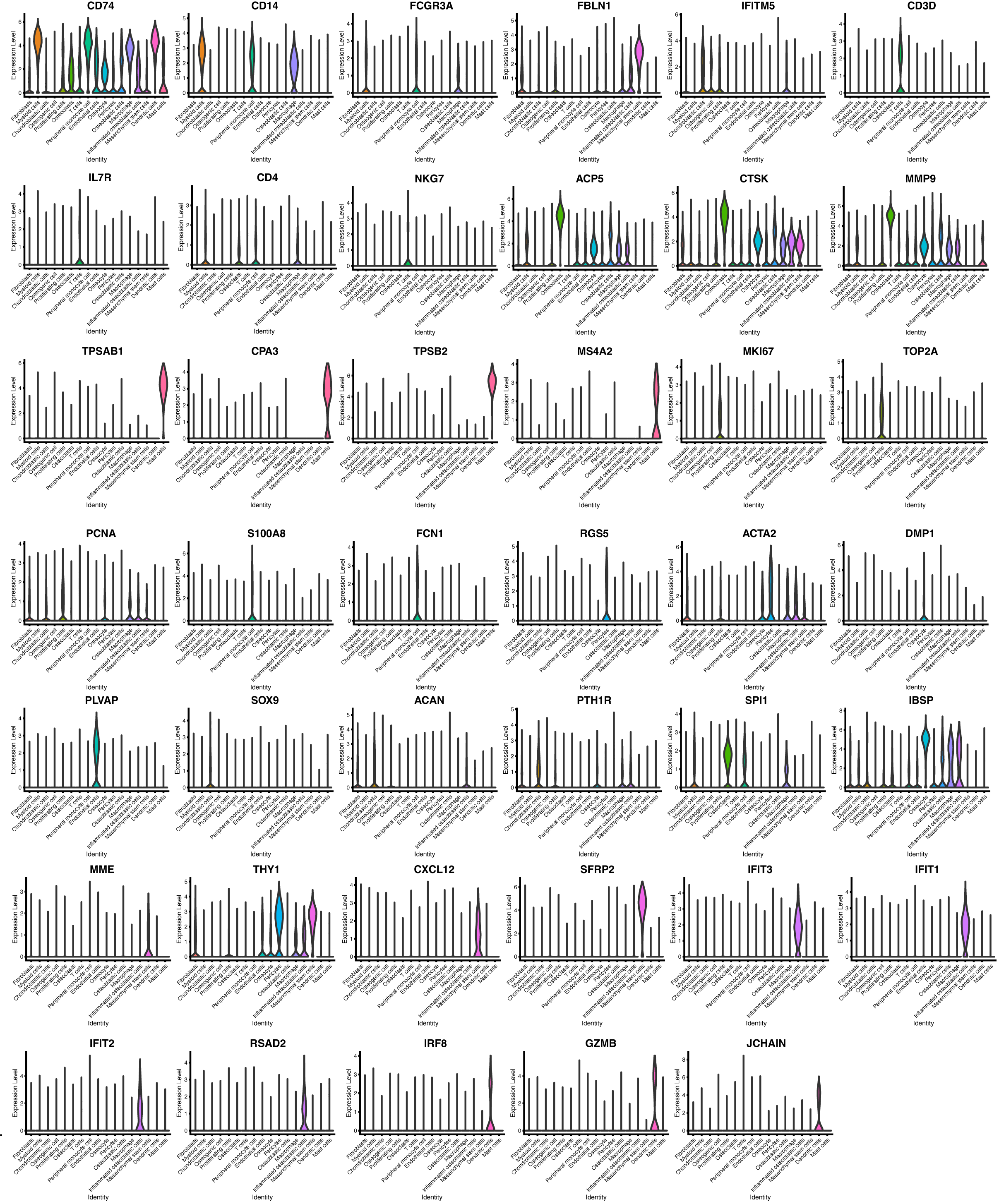

Supplement: Supplementary file 2 — Additional file 2: Supplementary Figure S2. Violin plots showed cell marker expression in various cell types of the scRNA-seq data. [file 12920_2023_1617_MOESM2_ESM.tif]

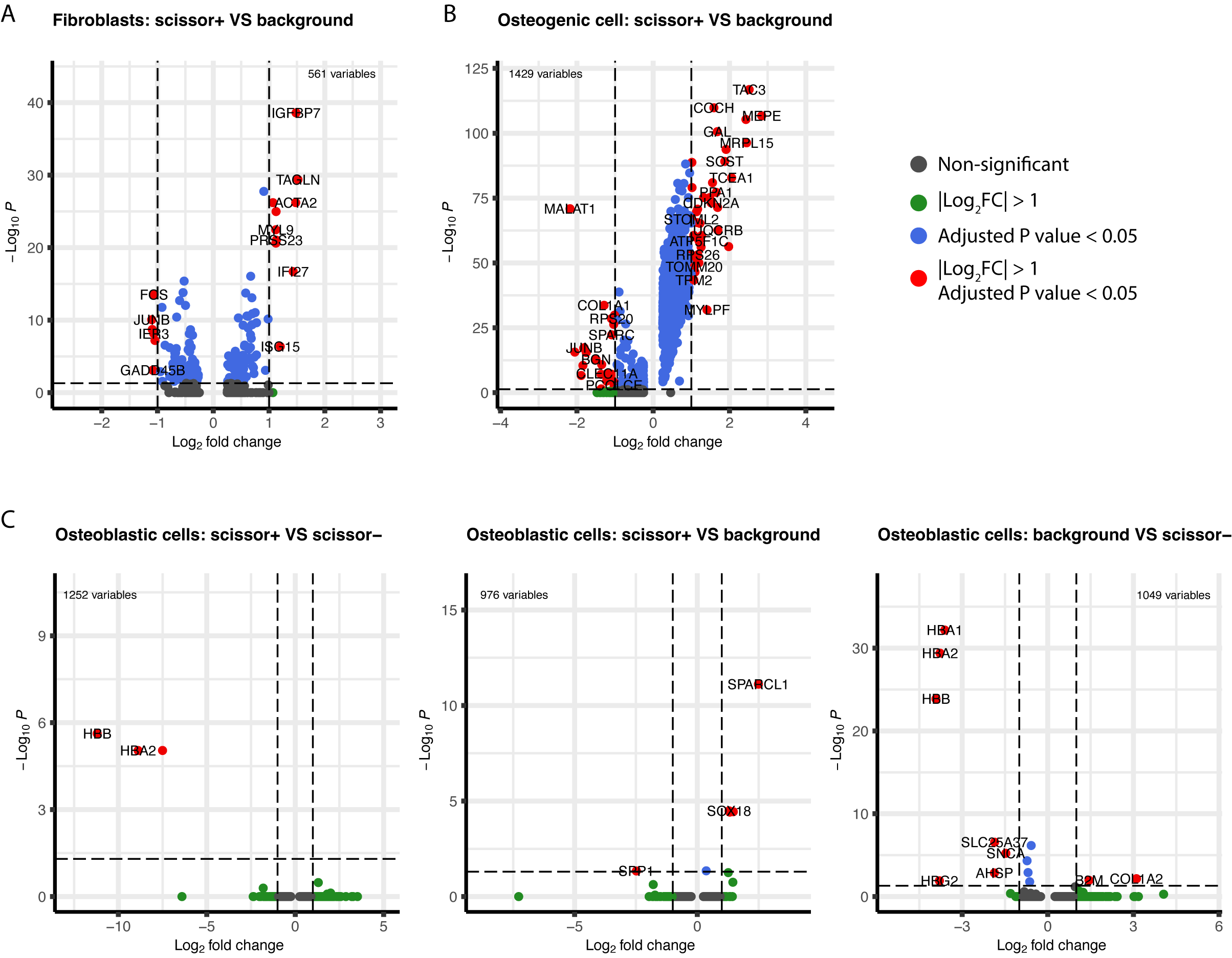

Supplement: Supplementary file 3 — Additional file 3: Supplementary Figure S3. The volcano plots of the differentially expressed genes considering fibroblasts (A), osteogenic cells (B), and osteoblastic cells (C). [file 12920_2023_1617_MOESM3_ESM.tif]

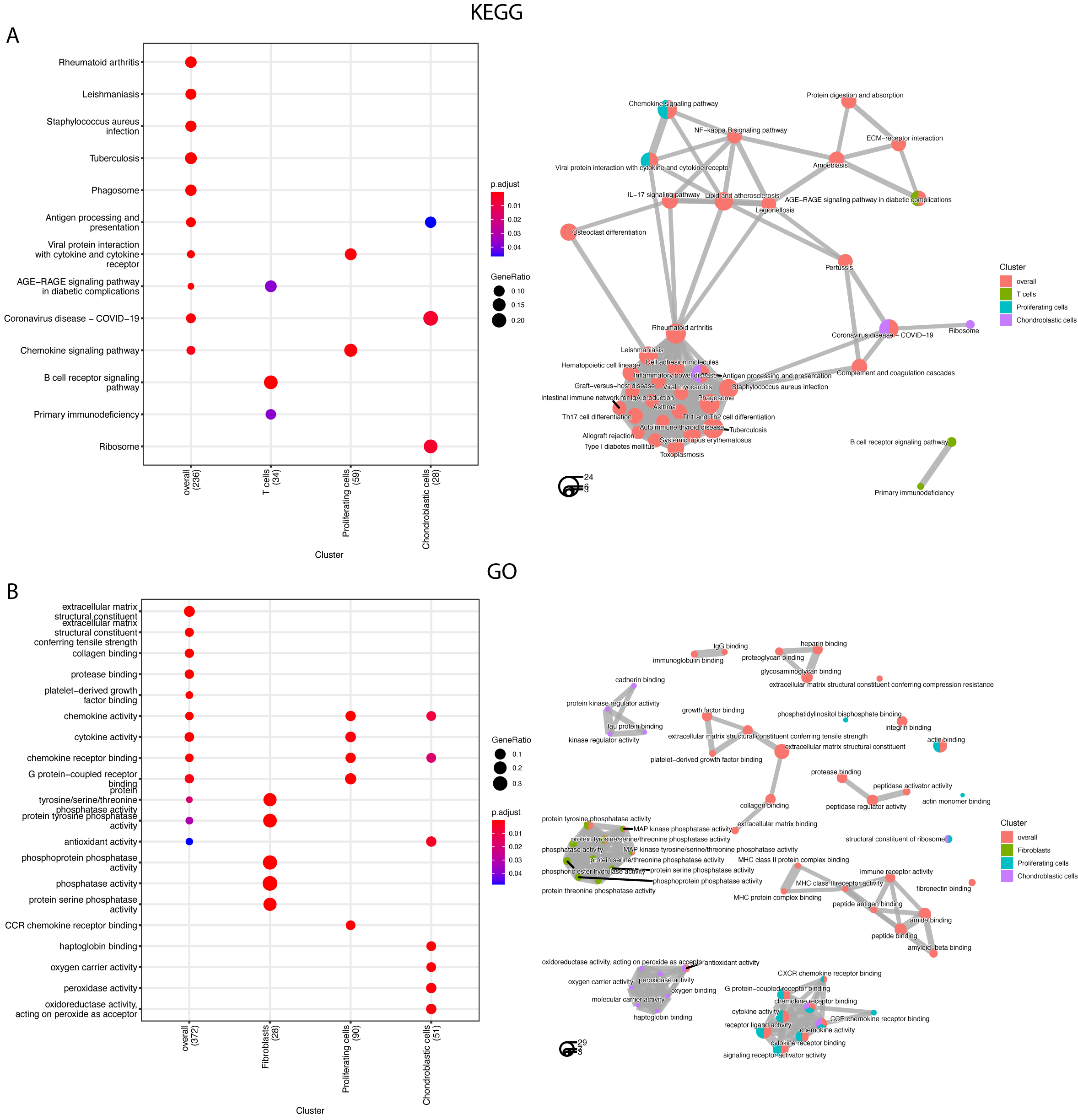

Supplement: Supplementary file 4 — Additional file 4: Supplementary Figure S4. Functional enrichments analysis of Kyoto Encyclopedia of Genes and Genomes (KEGG) pathways (A) and Gene Ontology (GO) terms (B). The dot-plot on the left panel represents the top five enriched items in KEGG or GO. The network on the right panel represents the connection between the KEGG pathways or GO terms. [file 12920_2023_1617_MOESM4_ESM.tif]

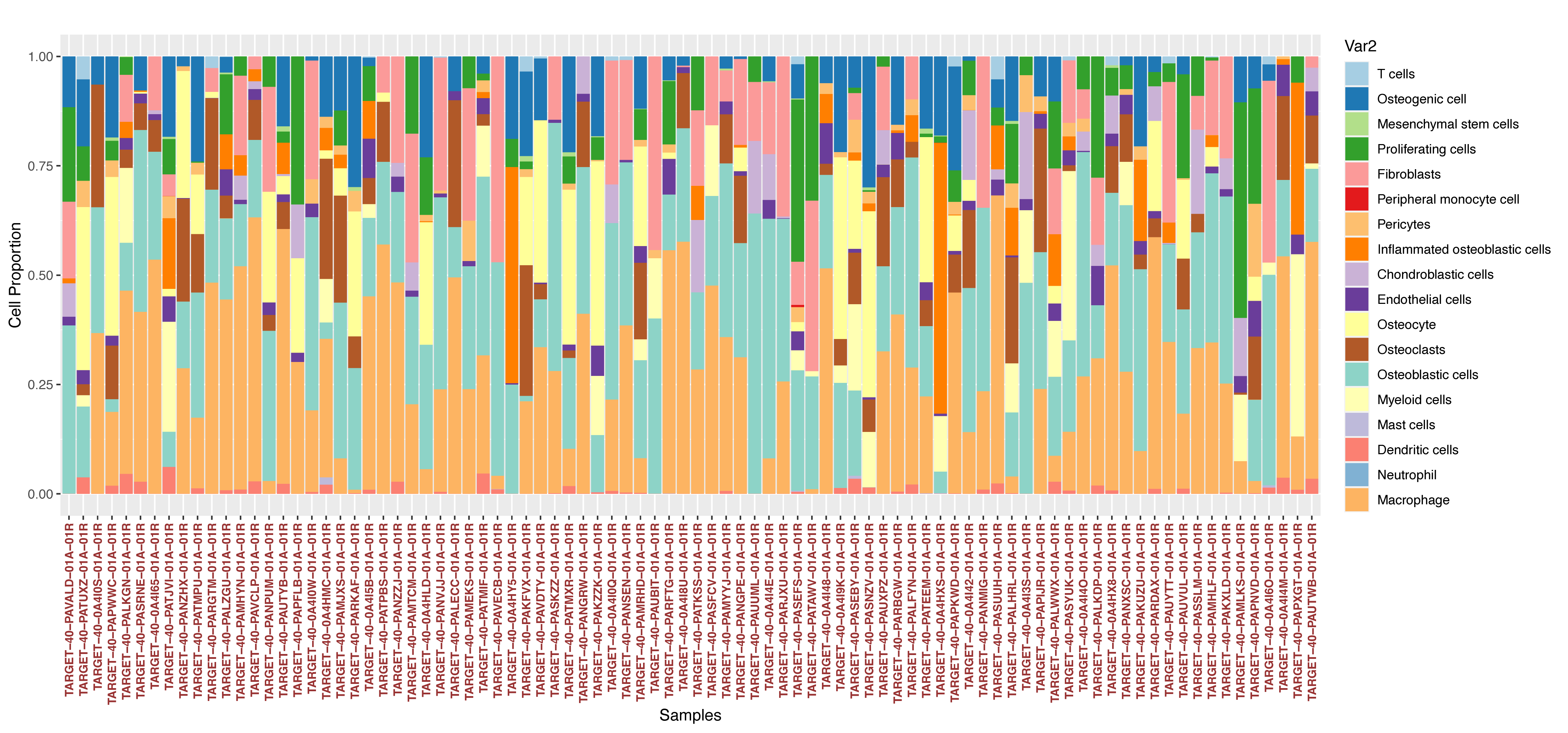

Supplement: Supplementary file 5 — Additional file 5: Supplementary Figure S5. Stacked bar-plot of cell proportion in the osteosarcoma bulk transcriptome data from TCGA. [file 12920_2023_1617_MOESM5_ESM.tif]
